# Checkpoint suppressor 1 suppresses transcriptional activity of ERα and breast cancer cell proliferation via deacetylase SIRT1

**DOI:** 10.1038/s41419-018-0629-3

**Published:** 2018-05-11

**Authors:** Zhaowei Xu, Yangyang Yang, Bowen Li, Yanan Li, Kangkai Xia, Yuxi Yang, Xiahui Li, Miao Wang, Shujing Li, Huijian Wu

**Affiliations:** School of Life Science and Biotechnology, Dalian University of Technology, Dalian, China

## Abstract

Breast cancer is a highly heterogeneous carcinoma in women worldwide, but the underlying mechanisms that account for breast cancer initiation and development have not been fully established. Mounting evidence indicates that Checkpoint suppressor 1 (CHES1) is tightly associated with tumorigenesis and prognosis in many types of cancer. However, the definitive function of CHES1 in breast cancer remains to be explored. Here we showed that CHES1 had a physical interaction with estrogen receptor-α (ERα) and repressed the transactivation of ERα in breast cancer cells. Mechanistically, the interaction between CHES1 and ERα enhanced the recruitment of nicotinamide adenine dinucleotide (NAD+) deacetylase Sirtuin 1 (SIRT1), and it further induced SIRT1-mediated ERα deacetylation and repression on the promoter-binding enrichment of ERα. In addition, we also found that the expression of CHES1 was repressed by estrogen-ERα signaling and the expression level of CHES1 was significantly downregulated in ERα-positive breast cancer. The detailed mechanism was that ERα may directly bind to *CHES1* potential promoter via recognizing the conserved estrogen response element (ERE) motif in response to estrogen stimulation. Functionally, CHES1 inhibited ERα-mediated proliferation and tumorigenesis of breast cancer cells in vivo and in vitro. Totally, these results identified a negative cross-regulatory loop between ERα and CHES1 that was required for growth of breast cancer cells, it might uncover novel insight into molecular mechanism of CHES1 involved in breast cancer and provide new avenues for molecular-targeted therapy in hormone-regulated breast cancer.

## Introduction

Estrogen signaling pathway is aberrantly active in hormone-responsive breast cancer, which has a vital role in the initiation and development of breast carcinoma^1,2^. Estrogen receptor-α (ERα), a member of nuclear receptor superfamily, serves as a key factor to regulate E_2_ response and signaling transduction^3,4^. In addition, it can promote estrogen-dependent cancer progression via regulating the transcription of genes linked to cell proliferation and survival^5,6^. ERα exhibits transcriptional activation or repression via recruiting co-activators or co-repressors to the promoters or enhancers of target genes, and the definitive function of ERα in transcriptional regulation is largely decided by its co-regulators in certain cellular context^7,8^. Although a large number of co-regulators of ERα have been identified, the integrated interaction network of ERα remains to be explored. Moreover, the function of ERα is also tightly associated with posttranslational modifications such as ubiquitination^2,9^, methylation^10^, phosphorylation^11^, acetylation^12,13^, and sumoylation^14,15^. Among these modifications, p300-mediated acetylation of ERα facilitates the E_2_-responsive DNA-binding ability and promotes its transcriptional activity^12^. Furthermore, ERα deacetylation is achieved by native cellular histone deacetylases (HDACs), such as Trichostatin A-sensitive enzymes (Class I and II HDACs) and nicotinamide-sensitive enzymes (Class III HDACs)^13^. Sirtuin 1 (SIRT1)-mediated deacetylation of ERα results in a repressive effect on ERα transactivation, which has a vital role in the progression of ERα-positive breast cancer^16^.

Forkhead box (FOX) proteins are a large transcriptional family which contains an evolutionary conserved forkhead or winged-helix DNA-binding domain^17^. They were identified in *Drosophila melanogaster* first^18^. In addition, there are more than 50 members found in human proteome and they are further categorized into 19 subgroups^18^. Despite studies that confirmed FOX proteins serve as key factors in embryogenesis, metabolism, and tumorigenesis, the molecular functions of FOX family members are divergent and even opposing^19,20^. Therefore, it warrants further investigation to explore the defined function of a certain FOX protein in different physiological processes. CHES1 (also named FOXN3) belongs to FOXN subgroup and contains a conserved FOX domain in N-terminal; it has a significant role in various processes including DNA damage^21,22^, cell cycle arrest^23,24^, and the development of organs^19,25^. In addition, CHES1 may act as a transcriptional repressor via interacting with SIN3A/HDACs complex^19,20,25^ or other co-regulators such as SKIP^26^, Menin^22^, and β-catenin^27^. A growing number of studies imply that CHES1 is tightly associated with tumor initiation and progression, and it is dysregulated in many types of carcinoma such as oral squamous cell carcinoma^28^, ovarian cancer^29^, colorectal cancer^27,30^, glioblastoma^31^, and hepatocellular carcinoma^32^. These studies have demonstrated that CHES1 had a weaker expression level in tumor tissues and its lower expression implied a poor prognosis^31^. Studies also show that CHES1 can inhibit protein biosynthesis^33^ or the transcriptional expression of oncogenes such as *E2F5*^32^, *c-MYC*^34^, and *CDH2*^30^. However, the defined function of CHES1 in breast cancer is still elusive. A recent study indicates that CHES1 may have a role in progress of metastasis and invasion in hormone-responsive breast carcinoma^20^, but more biological functions of CHES1 involved in breast cancer remain to be explored.

Herein, we identified the association between CHES1 and ERα in breast cancer cells; further investigation revealed that a negative regulatory loop between CHES1 and ERα existed in breast cancer, and this regulatory model modulated the signaling transduction of E_2_-ERα and had a role in progress and prognosis of breast cancer.

## Results

### CHES1 has physical interaction with ERα

**Fig. 1:**
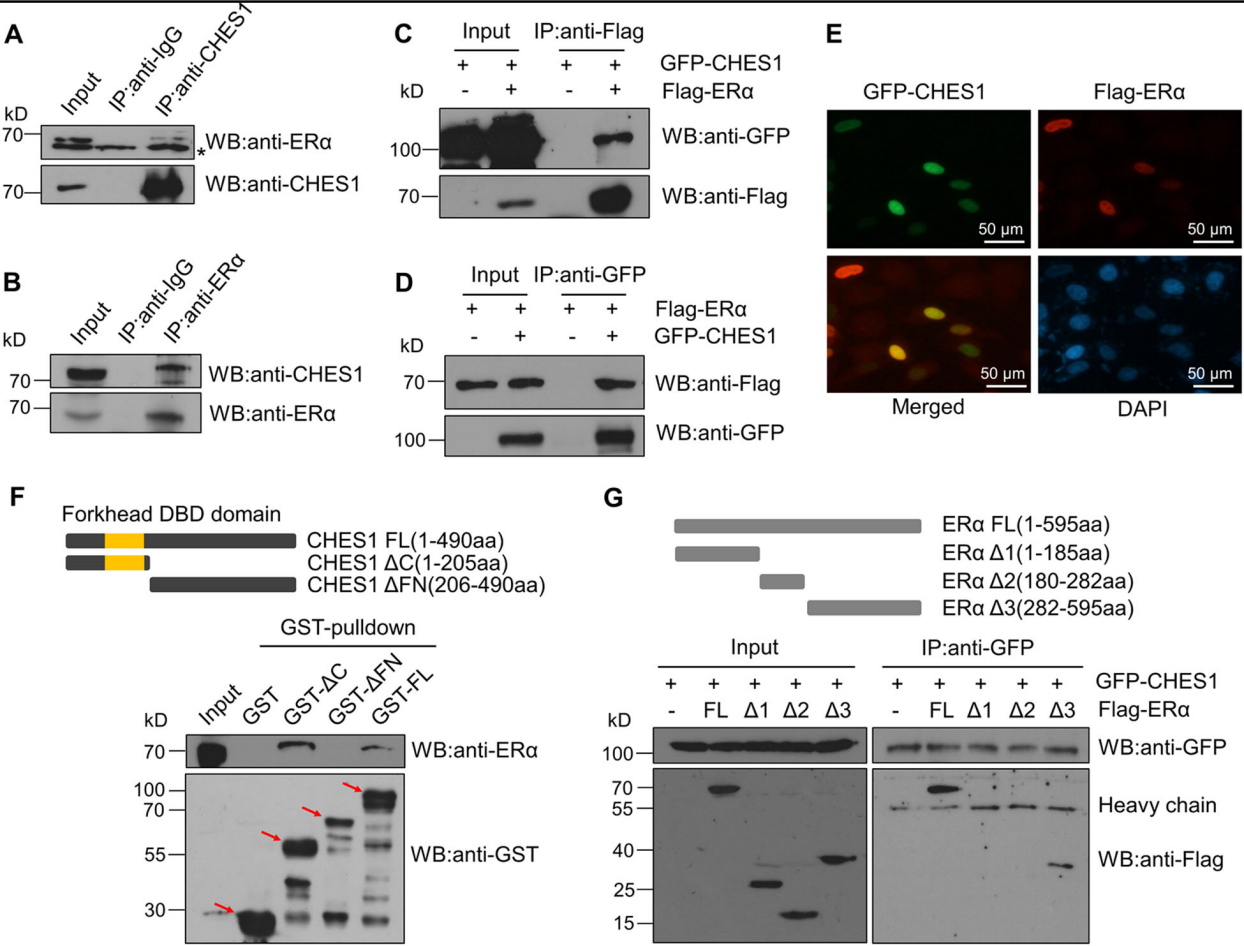
The interaction between CHES1 with ERα. **a**, **b** CoIP assay showed the interaction between endogenous CHES1 and ERα in MCF7 cells. **c**, **d** CoIP assay showed the interaction between exogenous CHES1 and ERα in HEK293T cells. **e** Immunofluorescence showed that CHES1 (green) and ERα (red) colocalized in in the nucleus (blue) of MCF7 cells. **f** GST-pulldown assays showed that purified GST-CHES1 had physical interaction with endogenous ERα in MCF7 cells and the FOX domain of CHES1 mediated their interaction. **g** CoIP assays showed that the residues 282–595 of ERα is required for the interaction between ERα and CHES1. *Nonspecific bands. The arrows indicate the positions of GST-tagged fused proteins we aimed to purify

As described previously, the other member of FOX protein family, FOXA1^35^, FOXO3a^36^, and FOXK2^37,38^, can modulate the activity of nuclear receptors via proteins physical interaction; here we speculated that CHES1, also known as FOXN3, may regulate ERα activity through the interaction with ERα in breast cancer cells. To confirm this possibility, we performed co-immunoprecipitation (CoIP) assay in MCF7 (Fig. 1a,b) and HEK293T cells (Fig. 1c,d). The results detected the existence of interaction between endogenous and exogenous CHES1 with ERα. To further support this assumption, immunofluorescence and (GST)-pulldown assays were conducted. The results showed that both GFP-CHES1 and Flag-ERα were located in the nucleus and an evident association was detected between purified Glutathione S-transferase GST-CHES1 and endogenous ERα in MCF7 cells (Fig. 1e,f).

Moreover, in order to validate which region of the two proteins mediated the interaction, several deletion mutants of CHES1 and ERα were constructed as described previously^33,39^. GST-pulldown and CoIP assays showed that the FOX domain of CHES1 and the residues 282–595 of ERα are required for their physical interaction (Fig. 1f,g). Taken together, these results indicate that a physical interaction between CHES1 and ERα may exist in breast cancer cells.

### CHES1 represses the transcriptional activity of ERα

**Fig. 2:**
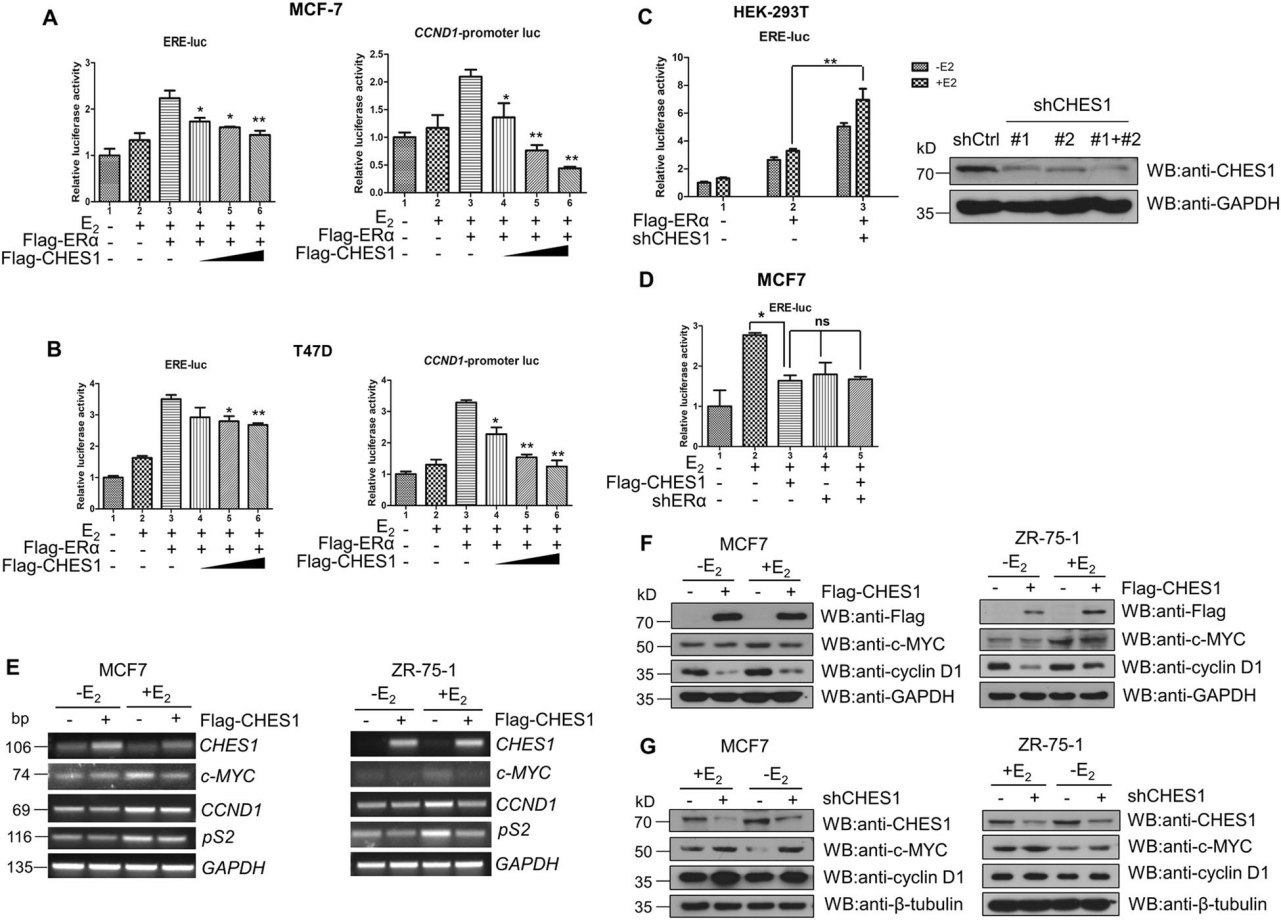
CHES1 repressed the transactivation of ERα. **a**, **b** Luciferase reporter assay showed that the transcription activity of ERα on ERE-luc and CCND1-promoter luc were repressed by CHES1 in MCF7 and T47D cells. **c** Luciferase reporter assay showed that the transactivation of ERα on ERE-luc was enhanced by shCHES1 in HEK293T cells. Knockdown of endogenous CHES1 by specific shRNA was tested by western blotting. **d** Luciferase reporter assay showed that the inhibitory effect of CHES1 on ERE-luc was diminished when endogenous ERα were knockdown by shRNA in MCF7 cells. Data are presented as means ± SD. *P < 0.05; **P < 0.01; ns, no significance. **e** RT-PCR assay detected the mRNA level of well-established ERα-target genes *c-MYC*, *CCND1*, and *pS2* with overexpression of Flag-CHES1 in MCF7 and ZR-75-1 cells. **f**, **g** Western blot assay tested the protein level of ERα-target genes c-MYC and Cyclin-D1 with forced expression of Flag-CHES1 or knockdown of endogenous CHES1 using shRNA in MCF7 and ZR-75-1 cells

To investigate the effect of the interaction between ERα and CHES1 on the transactivation of ERα, luciferase reporter assay was conducted in ERα-positive breast cancer cells. Overexpression of CHES1 attenuated the estrogen-dependent transcriptional activity of ERα in MCF7 and T47D cells when using ERE-luc and *CCND1*-promoter luc (Fig. 2a,b). Then the similar assay was performed in ERα-negative/CHES1-high expressing HEK293T cells. Knockdown endogenous CHES1 by short hairpin RNA (shRNA) significantly enhanced the transactivation of Flag-ERα (Fig. 2c). To further confirm the transcriptional repression of CHES1 on ERα transactivation depended on ERα, stably knockdown of ERα was performed in MCF7 cells as described previously^40^. Luciferase reporter assay showed that the repression effect of CHES1 was diminished when ERα expression was interfered (Fig. 2d). Together, those results indicate that CHES1 may specifically repress ERα-mediated transcriptional activity in ERα-positive breast cancer cells.

To further consolidate the observation, we tested the inhibitory effect of CHES1 on the expression of well-established ERα target genes (*CCND1*, *c-MYC*, and *pS2*) in MCF-7 and ZR-75-1 cells. Further results showed that overexpression of CHES1 attenuated both mRNA and protein levels of these genes (Fig. 2e,f). Reciprocally, knockdown of endogenous CHES1 enhanced the protein level of Cyclin D1 and c-MYC (Fig. 2g). Together, these data support a notion that CHES1 may repress the transactivation of ERα via physical interaction in ERα-positive breast cancer cells.

### CHES1 enhances SIRT1-mediated deacetylation of ERα and repression on ERα activity

**Fig. 3:**
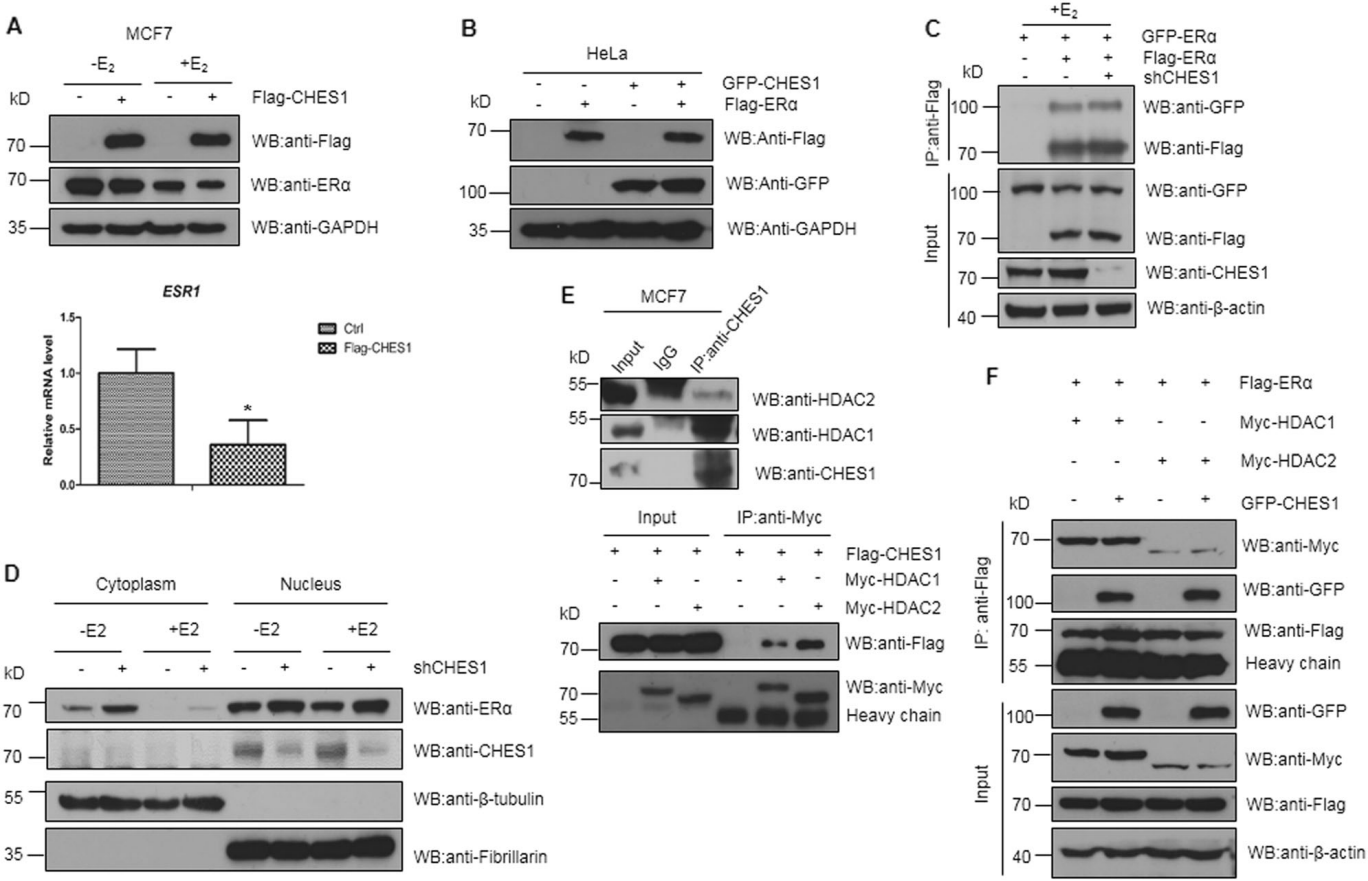
CHES1 has little effect on the stability, dimerization, subcellular location of ERα, and it also does not influence the interaction between ERα and HDAC1/2. **a** Western blotting and real-time PCR assays tested the mRNA and protein level of endogenous ERα with overexpression of Flag-CHES1 in MCF7 cells. **b** Western blotting assay showed that the protein level of exogenous ERα were not affected when co-transfected with CHES1 in HeLa cells. **c** CoIP assay indicated that the dimerization of ERα were not interfered with knockdown of CHES1 in HEK293T cells. **d** Cytoplasmic and nucleus fractions separation assay were conducted in MCF7 cells with or without knockdown of CHES1 to test the subcellular distribution of endogenous ERα. The β-tubulin used as cytoplasmic marker and Fibrillarin as nuclear marker. **e** CoIP assay showed the endogenous and exogenous interaction between CHES1 and HDAC1/2 in MCF7 and HEK293T cells. **f** CoIP assay assessed the effect of CHES1 on the interaction between ERα and HDAC1/2

To further establish the detailed mechanism that the inhibitory effect of CHES1 on the transactivation of ERα, we first tested whether CHES1 had influence on transcriptional expression of ERα. The results showed that the mRNA and protein levels of ERα were repressed with overexpression of Flag-CHES1 in MCF7 cells (Fig. 3a); these results consist with the study reported by others^20^. However, considering the existence of interaction between CHES1 and ERα, and how this interaction affect ERα activity remains to be illuminated. Therefore, we first tested whether the protein stability of ERα was affected by CHES1, because FOXK2, another member of FOX family, was established to promote degradation of ERα^37^. Co-transfection with Flag-ERα and GFP-CHES1 in HeLa cells showed that the ERα stability was not significantly changed by CHES1 (Fig. 3b). As ERα exhibits its function via forming homodimer in response to E_2_ stimulation and then transferring into nuclear, we first tested whether the formation of ERα homodimer was disturbed by CHES1. The result showed that CHES1 did not have a significant effect on ERα dimerization (Fig. 3c). Moreover, the subcellular location of ERα was tested with knockdown of CHES1. Cytoplasmic and nuclear fractionation and immunofluorescence assays showed that CHES1 had little effect on the subcellular distribution of ERα in MCF7 cells (Fig. 3d and Figure S1A). It is commonly known that HDAC1 and HDAC2 both act as co-repressors of ERα and CHES1 could also recruit HDAC1/2 complex to achieve transcriptional suppressing function; it is necessary to elucidate that whether the interaction between HDAC1/2 and ERα were interfered by CHES1. To demonstrate this possibility, the interaction between CHES1 and HDAC1 or HDAC2 were confirmed by IP assay first (Fig. 3e). Then another IP assay indicated that CHES1 had little effect on the interactions between ERα and HDAC1/2 (Fig. 3f).

**Fig. 4:**
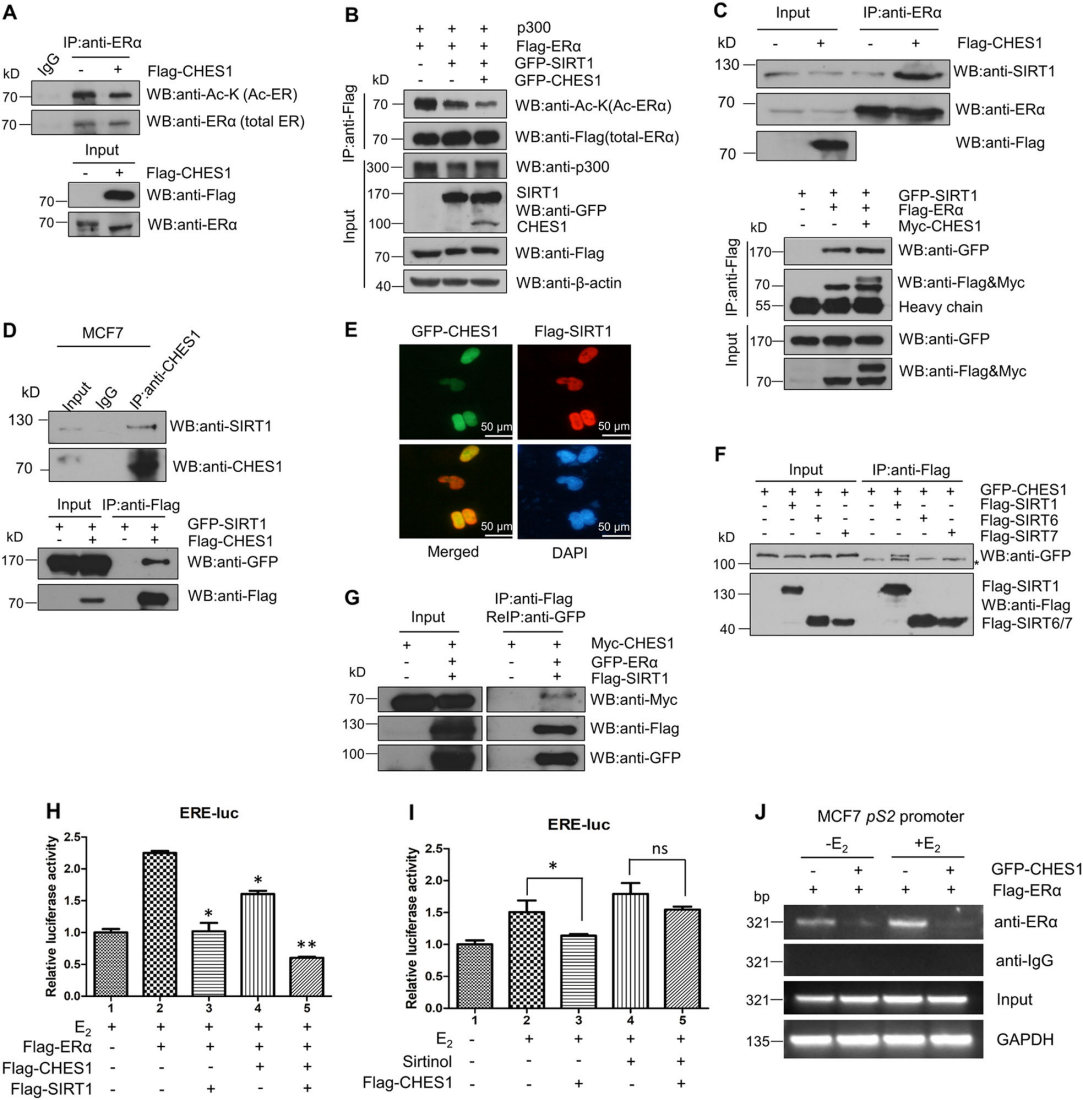
CHES1 enhanced the recruitment of SIRT1 and SIRT1-mediated deacetylation and transrepression on ERα activity. **a** With the treatment of 1 μM deacetylase inhibitors Trichostatin A (TSA) and 100 nM E_2_ for 24 h, CoIP assay tested the endogenous acetylation level of ERα with or without overexpression of CHES1 in MCF7 cells. **b** CoIP assay tested the exogenous acetylation level of ERα with overexpression with CHES1 and SIRT1 in HEK293T cells. The relative protein level was normalized with β-actin. **c** CoIP assay showed that CHES1 enhanced the recruitment of SIRT1 to ERα in MCF7 and HEK293T cells. **d** CoIP assay showed that the endogenous and exogenous interaction between CHES1 and SIRT1 in MCF7 and HEK293T cells. **e** Immunofluorescence showed that CHES1 (green) and SIRT1 (red) colocalized in the nucleus (blue) of MCF7 cells. **f** CoIP assay showed that CHES1 only interacted with SIRT1 but not SIRT6 and SIRT7. *Nonspecific bands. **g** IP/ReIP assay tested the existence of the complex of ERα/CHES1/SIRT1. **h** Luciferase reporter assay showed that CHES1 enhanced the repression of SIRT1 on transactivation of ERα. **i** Luciferase reporter assay demonstrated that the inhibitory effect of CHES1 on transcription activity of ERα was diminished in MCF7 cells when treated with Sirtinol (the SIRT1 inhibitor). **j** ChIP assay conducted in MCF7 cells indicated that the repressive effect of CHES1 on the enrichment of ERα in the *pS2* promoter

As the HDACs can also deacetylate non-histone proteins, the dynamic acetylation and deacetylation of non-histone proteins are in charge of various proteins activity^41,42^. It has been confirmed that the transactivation of ERα is coupled with acetylation and deacetylation mediated by p300^12,13^ and deacetylase such as SIRT1^43,44^. As we have proved that CHES1 could repress the transactivation of ERα and also exhibit its function via recruiting HDACs^20,45^, it proposed that CHES1 may regulate ERα acetylation to achieve transcriptional repression via recruiting deacetylases SIRT1. To support this possibility, the acetylation level of endogenous and exogenous ERα were tested by IP assays in MCF-7 and HEK293T cells. The data showed that overexpression of CHES1 attenuated the acetylation level of ERα (Fig. 4a,b). Then we speculated CHES1 may enhance the recruitment of the deacetylase SIRT1 to ERα and then induce SIRT1-mediated deacetylation of ERα. To test this notion, IP assay was conducted and the results showed that CHES1 could enhance the endogenous and exogenous interaction between ERα and SIRT1 (Fig. 4c).

To further consolidate this possibility, the interaction between CHES1 and SIRT1 was confirmed by IP, immunofluorescence, and GST-pulldown assays (Fig. 4d,e and S1D). In addition, further IP assay indicated that CHES1 can only interact with SIRT1 but not with other nucleus located Sirtuins (SIRT6 or SIRT7) (Fig. 4f). Given that CHES1 could both interact with SIRT1 and ERα, we speculated that an ERα-CHES1-SIRT1 complex might exist. To investigate this notion, IP/ReIP assay was conducted and the positive band was detected (Fig. 4g). The luciferase reporter assay also indicated that CHES1 enhanced SIRT1-mediated repression on ERα transactivation (Fig. 4h). To further confirm that CHES1 repressed ERα-mediated transcription activity via deacetylase SIRT1, Sirtinol, the selective inhibitor of SIRT1^46^, was introduced in luciferase reporter assay. The repressive ability of CHES1 on ERE-luc diminished when the activity of SIRT1 has been inhibited by Sirtinol (Fig. 4i). Given that ERα acetylation enhances its DNA-binding activity and transactivation, chromatin immunoprecipitation (ChIP) assay was conducted to demonstrate the effect of CHES1 on the DNA-binding enrichment of ERα at *pS2* promoter in MCF7 cells. The result showed that the abundance of ERα in the promoter was attenuated when overexpressed CHES1 (Fig. 4j). Totally, these results indicate CHES1 may inhibit ERα transactivation and promoter occupancy enrichment via enhancing SIRT1 recruitment and the SIRT1-mediated deacetylation of ERα.

### CHES1 expression is suppressed by E_2_-ERα in breast cancer

**Fig. 5:**
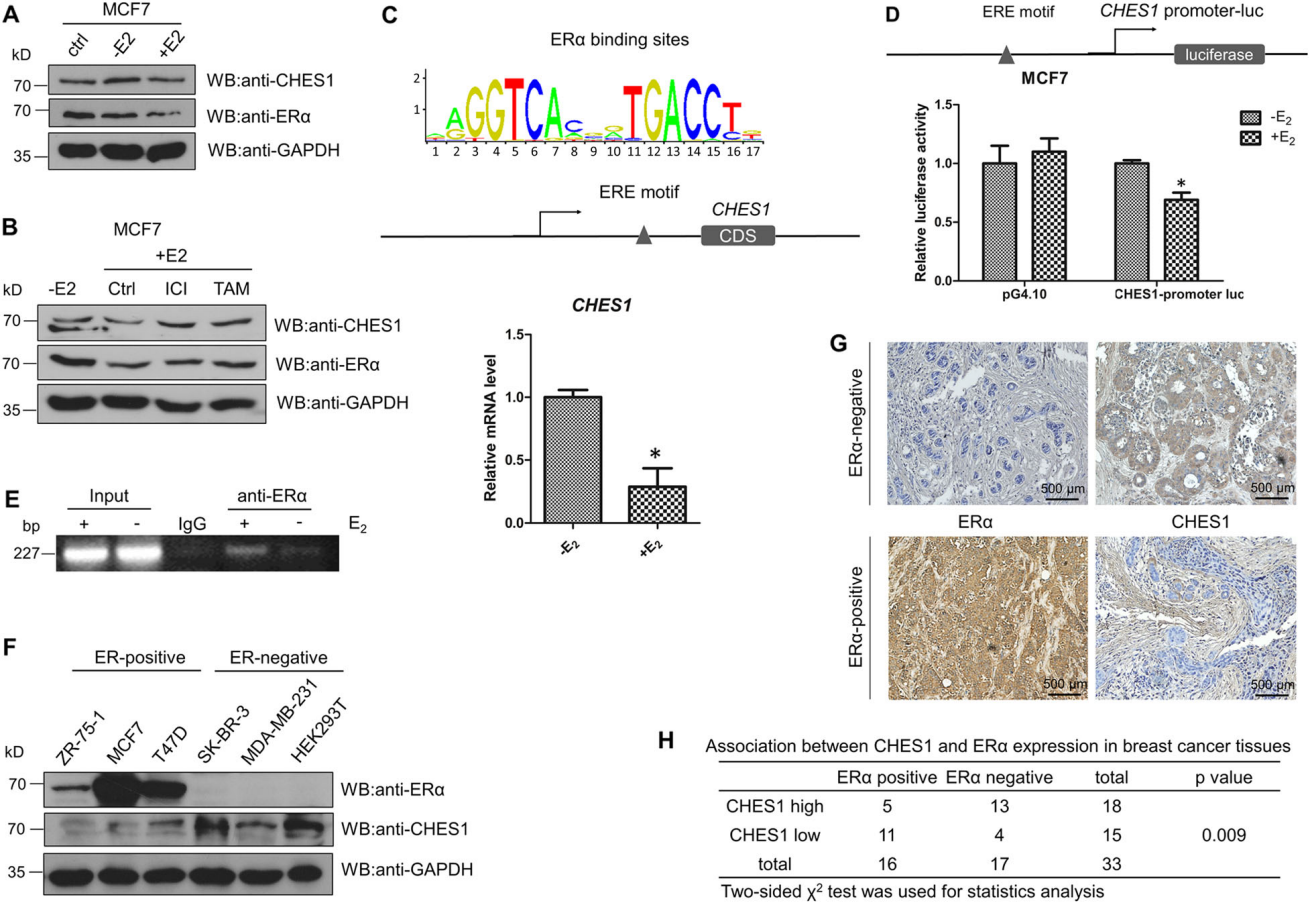
CHES1 expression was repressed by E_2_-ERα pathway in breast cancer. **a** Western blotting tested the protein expression of CHES1 and ERα in MCF7 cells with or without E_2_ treatment. **b** Immunoblotting of CHES1 and ERα in MCF7 cells treated with or without 100 nM E_2_, 1 nM 4-Hydroxytamoxifen, and 0.1 μM ICI 182780. **c** Schematic representation of conserved ERα-binding motif and ERE site on *CHES1* potential promoter region, real-time PCR assay tested the mRNA level of *CHES1* in MCF7 cells treated with or without E_2_. **d** Schematic representation of *CHES1*-promoter luc construction. Luciferase reporter assay showed that the activity of *CHES1*-promoter luc was repressed in MCF7 cells when treated with E_2_ stimulation. *P < 0.05. **e** ChIP assay conducted in MCF7 cells detected the enrichment of ERα binding on ERE motif in *CHES1* promoter. **f** Western blotting assay detected the expression level of ERα and CHES1 in ERα-positive and ERα-negative cell lines. **g** The representative images showed IHC staining the protein level of CHES1 and ERα in breast cancer patient tissues. **h** Correlation between ERα and CHES1 expression in breast cancer tissues. Two-sided *χ*^2^-test was used for statistics analysis

As described previously, genechips data identified that the expression of *CHES1* was decreased in ERα-positive cells MCF7 and ERα stably expressing MDA-MB-231 cells when treated with E_2_^47^; these results indicate a possibility that *CHES1* may be regulated by E_2_-ERα signaling in breast cancer cells. To verify this hypothesis, CHES1 expression was detected in MCF7 cells treated with or without E_2_. As results shown, endogenous CHES1 was decreased in response to E_2_ treatment (Fig. 5a). To further consolidate this possibility, 4-Hydroxytamoxifen (an E_2_ agonist) and ICI 182780 (an ERα specific inhibitor) were introduced to antagonize E_2_ stimulation. Consistently, the drugs reversed the inhibitory effect of E_2_-ERα on the expression of CHES1 (Fig. 5b). As shown in Fig. 3b, ERα may have little effect on the stability of CHES1, so the inhibitory effect of E_2_-ERα on CHES1 may be achieved via transcriptional level. Then, real-time PCR results confirmed that *CHES1* mRNA level was largely decreased in ERα-positive breast cancer cells MCF7 when responding to E_2_ stimulus (Fig. 5c). Moreover, a conserved ERE was found at − 2048 to − 2064 bp region upstream of the Coding sequence (CDS) in potential promoter of *CHES1* (Fig. 5c). Then, the *CHES1*-promoter luc covered 1000 bp DNA fragment containing the conserved ERE was constructed using pGL4.10 vector. MCF7 cells transfected the construct showed a significantly decreased luciferase activity when treated with E_2_, but similar effect was not detected when using the control vector (Fig. 5d). In addition, the binding of ERα on this region was confirmed by ChIP assay (Fig. 5e).

Furthermore, the expression levels of CHES1 and ERα in different cell lines were tested by western blotting. The results showed that ERα-positive breast cancer cells owned a lower level of CHES1, whereas ERα-negative breast cancer cells had a higher level (Fig. 5f). To extend our observations to clinicopathologically relevant contexts, 16 ERα-positive and 17 ERα-negative breast carcinoma samples were collected to analyze the protein levels of CHES1 and ERα by immunohistochemical staining assay. Consistently, the results and statistics analysis showed that a significantly reverse association between CHES1 and ERα existed in breast cancer tissues (Fig. 5g,h). To further confirm this observation, analysis of a public dataset Oncomine (https://www.oncomine.org/resource/login.html) also revealed that ERα-positive breast cancer showed a lower level of CHES1 and ERα-negative breast cancer owned a higher one (Figure S2). Together, these results reveal that CHES1 expression may be repressed by E_2_-ERα in breast cancer.

### CHES1 inhibits proliferation and tumorigenesis of ERα-positive breast cancer cells

**Fig. 6:**
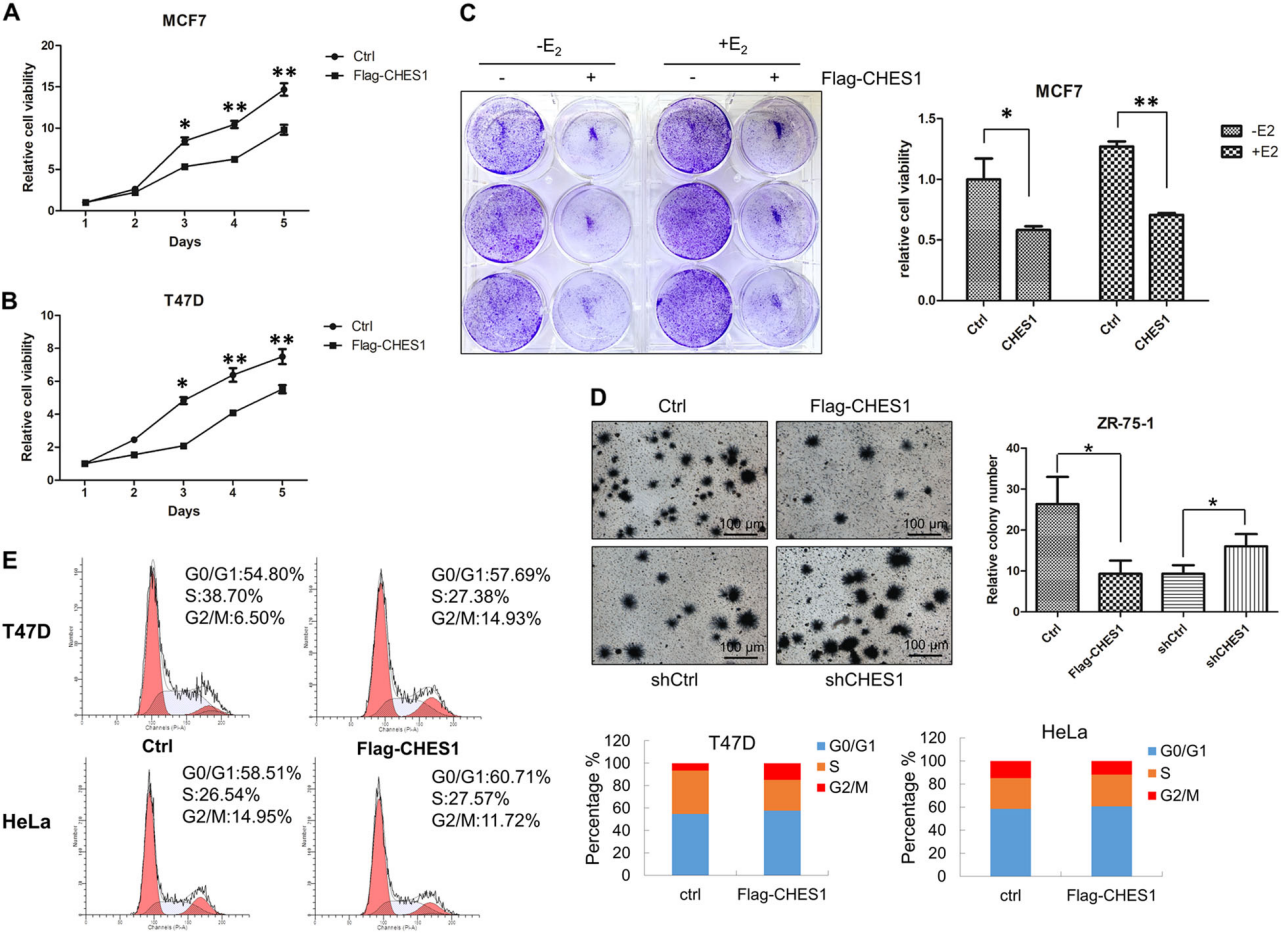
CHES1 inhibits proliferation of ERα-positive breast cancer. **a**, **b** The growth curves of MCF7 and T47D cells with or without stably overexpression of Flag-CHES1 were measured with CCK8 assay. **c** Crystal violet staining assay evaluated the ability of colony formation of MCF7 cells with stably expressing Flag-CHES1 or control vector. **d** The soft-agar colony culture assay was conducted to test the anchorage independent growth activity of ZR-75-1 cells. **e** Flow cytometry analysis was performed to test the cell cycle distribution of T47D and HeLa cells stably transfected with Flag-CHES1 or control vector

**Fig. 7:**
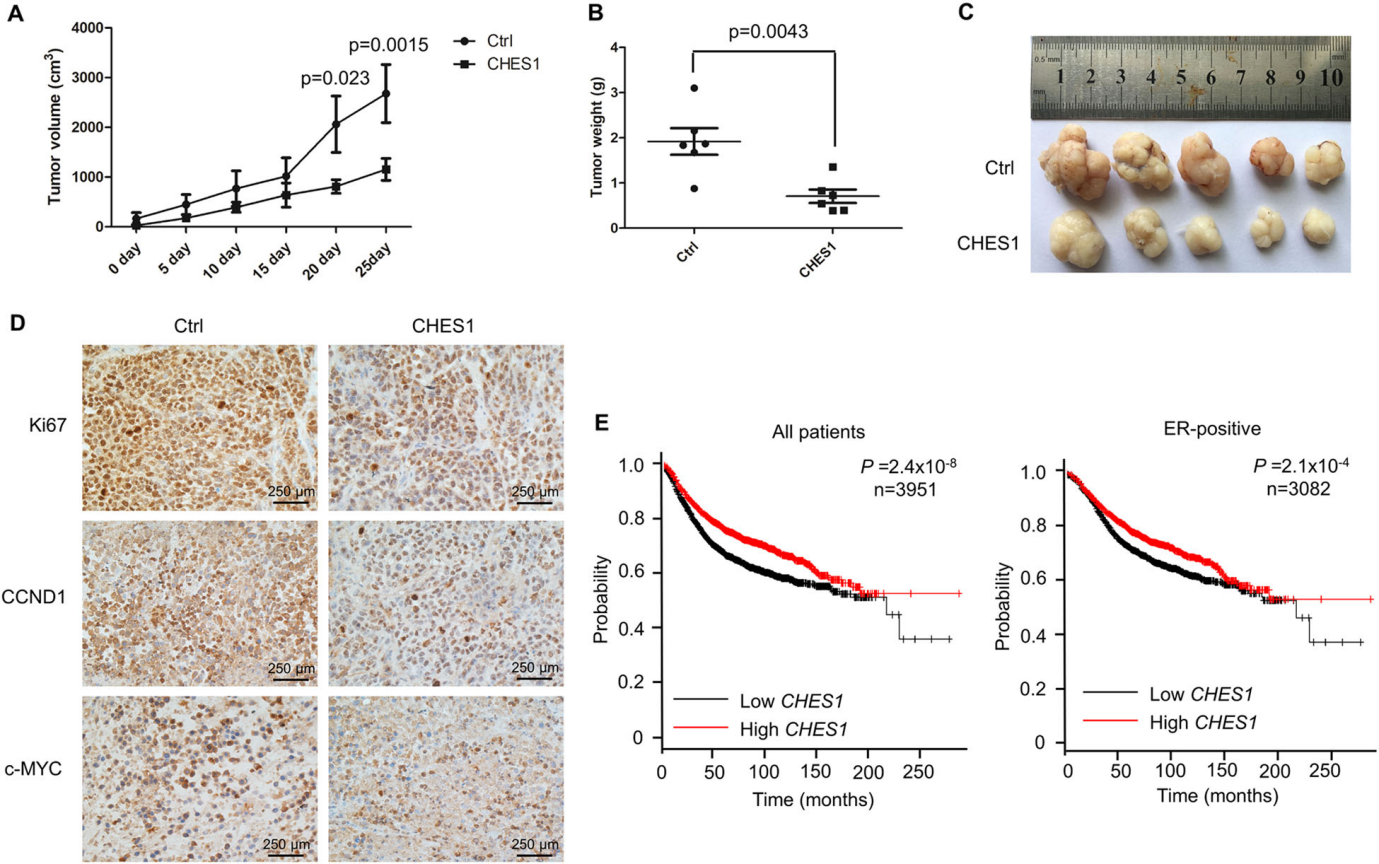
CHES1 inhibits tumorigenesis of ERα-positive breast cancer in vivo and high expression level of CHES1 indicates a better prognosis. Stably transfected MCF7 cells with Flag-CHES1 showed a significant reduction of tumor volume (**a**) and tumor weights (**b**) compared with control group. **c** The images of tumors were shown to compare the tumor size of each groups (*n* = 5 per group). *P < 0.05, **P < 0.01. **d** IHC staining assay showed the expression levels of CCND1, c-MYC, and Ki67 in tumors dissected from nude mice. **e** Kaplan–Meier curves of relapse-free survival times of total breast cancer patients (*n* = 3951) and patients with ERα-positive breast cancer (*n* = 3082), stratified by *CHES1* expression levels. Data were obtained from http://kmplot.com/analysis/. Statistical significance was determined by the log-rank test

As ERα is required for the proliferation of hormone-responsive breast cancer cells, the association between CHES1 and ERα may have a role in ERα-mediated growth of breast carcinoma. To test this hypothesis, cell proliferation assay was conducted in ERα-positive breast cancer cells MCF7 and T47D. The results indicated that the growth activities of MCF7 and T47D cells were significantly repressed with overexpression of Flag-CHES1 (Fig. 6a,b) and knockdown of CHES1 enhanced the cell viability (Figure S4C). To further consolidate this observation, colony formation and soft-agar colony culture assays were also performed, and the results showed that overexpression of CHES1 resulted in a marked decrease in colony formation number and knockdown of CHES1 increased the colony formation (Fig. 6c,d). In addition, cell cycle assay also demonstrated that the cell cycle of T47D cells was arrested with ectopic expression of CHES1, whereas the cell cycle process of ERα-negative cells HeLa was not affected (Fig. 6e). These data indicated that CHES1 may specifically repress ERα-mediated proliferation and cell cycle process in vitro. Moreover, human breast cancer xenograft mouse model was constructed to investigate the role of CHES1 in tumorigenesis of breast cancer in vivo. Host with stable expression of Flag-CHES1 exhibited much smaller size in tumor volume and tumor weight compared with the control group (Fig. 7a-c). In addition, we further tested the expression level of ERα-target genes (CCND1 and c-MYC) and proliferation biomarker Ki67 in these tumors. The immunohistochemistry (IHC) staining results confirmed that CHES1 significantly repressed the ERα target genes expression and inhibited the growth ability of breast cancer cells (Fig. 7d). Bioinformatics analysis also showed that CHES1 expression was significantly downregulated in breast cancer tissues compared with normal ones, which indicated CHES1 may act as a tumor suppressor in breast cancer (Figure S3)^48^. More importantly, we also evaluated the association between CHES1 level and the prognosis of breast cancer patients from a microarray data set of breast tumors^49^. Among 3951 total breast cancer and 3082 ERα-positive breast cancer patients, the patients with high CHES1 expression level had much longer relapse-free survival time than those with low level (Fig. 7e). Together, these results indicated that CHES1 exhibits an inhibitory effect on the proliferation and tumorigenesis potential of ERα-positive breast cancer cells in vitro and in vivo, and the high level of CHES1 may indicate a better prognosis.

## Discussion

Studies have confirmed that ERα acts as a key regulator for hormone-responsive breast cancer and is required for the growth of ERα-positive breast cancer. In addition, clinical researches have demonstrated that ERα is one of the most successful molecular targets for drug therapy^1^. However, the comprehensive regulatory network of ERα in breast cancer has not been fully elucidated. Herein, we demonstrated that CHES1 could interact with ERα through its evolutionary conserved forkhead domain. Furthermore, CHES1 could repress ERα-mediated transactivation in breast cancer cells but had little effect on the stability, cellular location and dimerization of ERα. Mechanistically, further data indicated that CHES1 could enhance the recruitment of deacetylase SIRT1 and promote SIRT1-mediated deacetylation of ERα. Functionally, CHES1 formed a complex with ERα and SIRT1 and then decreased the acetylation level and promoter-binding enrichment of ERα. In addition, we also detected a decrease mRNA and protein level of ERα with ectopic expression of CHES1 in MCF7 cells. This data consisted with a study reported recently that CHES1 could repress the transcriptional expression of *ESR1* via an HADC1/2-dependent way^20^. Considering that, CHES1 may regulate the activity of ERα through two ways in breast cancer cells. One is that CHES1 repressed the transcriptional expression of ERα in an HDAC1/2-dependent way; on the other hand, CHES1 directly interacted with ERα and then facilitated SIRT1-mediated deacetylation of ERα without involving HDAC1/2. Furthermore, our work also revealed that CHES1 not only interacted with class I HDAC1 and HDAC2, but also had association with class III deacetylase SIRT1. Which one CHES1 prefer to interact with or CHES1 could interact with all of these deacetylases simultaneously is also remained to be investigated. However, considering that the nuclear receptors can engage in multiple nuclear complexes to exhibit diverse actions of gene regulation^50^, an alternative explanation is that CHES1 acts as the co-regulator of ERα and participates in different transcriptional complexes, which are composed of different classes of deacetylase. Taken together, our study introduces an alternative model that the ERα transcriptional activity is regulated by CHES1 in breast cancer cells and reveals a novel way of CHES1 involved in transcriptional regulation.

**Fig. 8:**
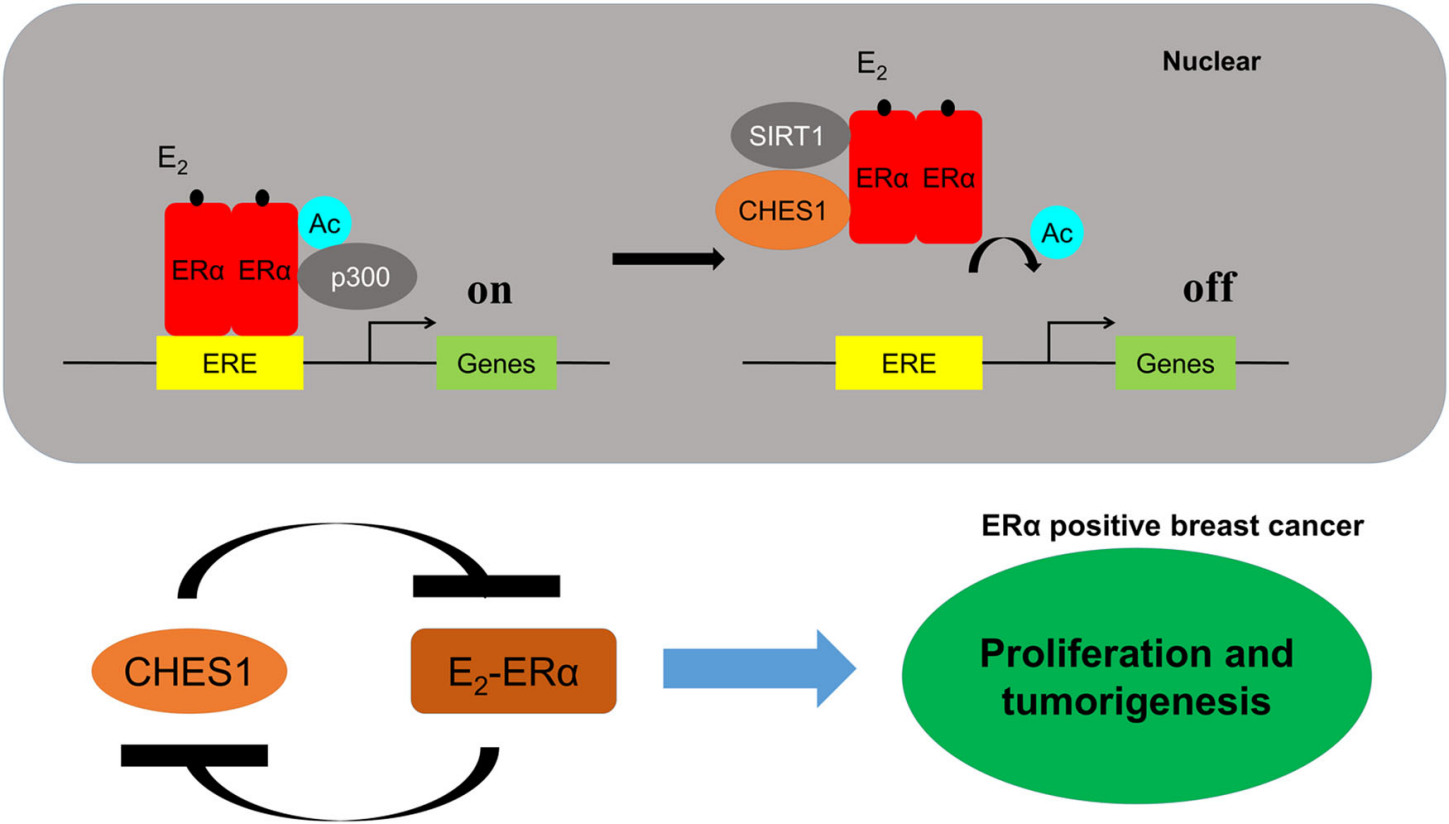
The schematic representation of negative regulatory loop between ERα and CHES1 in breast cancer

Previous studies have reported that CHES1 expression is downregulated in many types of carcinoma and its expression level has tight association with malignancy progression and prognosis. Here we found that the CHES1 expression was repressed in ERα-positive breast cancer cells when treated with E_2_. Furthermore, we explored the molecular mechanism involved and identified a conserved ERE in the regulatory region of *CHES1* promoter. Our results confirmed the possibility that E_2_-ERα signaling pathway may have an inhibitory effect on the transcriptional expression of *CHES1* and ERα could directly bind to the promoter of *CHES1* by recognizing the ERE motif and further repress *CHES1* transcription. Consistently, a negative association between ERα and CHES1 expression was identified in breast cancer cell lines and tumor tissues. In addition, we also confirmed this finding in public databases. Previous study and our results confirmed that CHES1 also reversely transrepressed ERα expression in ERα-positive breast cancer^20^; hence, a negative regulatory loop between ERα and CHES1 may exist in ERα-positive breast cancer (Fig. 8).

As previous studies indicated, CHES1 may act as a tumor-repressing protein in multiple cancer types such as colorectal carcinoma^30^, hepatic carcinoma^32^, glioblastoma^31^, and oral squamous cell carcinoma^28^. Consistently, the study here revealed that CHES1 could inhibit ERα-mediated proliferation and tumorigenesis through physical association with ERα in breast cancer cells. However, a study showed that CHES1 promoted epithelial-to-mesenchymal transition (EMT) in hormone-responsive breast cancer^20^, which indicated CHES1 may exhibit oncogenic functions. However, EMT is relatively uncorrelated and independent with proliferation processes, and the gene expression profile and regulatory network of EMT are largely far from growth and proliferation^51^. Furthermore, many molecules and signaling pathways may serve as dual roles in the processes of growth and EMT^43,44,52,53^, such as transforming growth factor-β1^52,53^ and SMADs^54^. Therefore, the role of CHES1 had in EMT and growth may be tightly dependent on signaling crosstalk or different partners in specific cellular environment.

In the present study, we revealed that CHES1 may have a role in ERα-positive breast cancer, but the defined biological role of CHES1 in other subtypes of breast cancer has not been fully elucidated. We also found that high CHES1 expression level indicated a better prognosis in patients with ERα-negative breast cancer (Figure S4F). In addition, the interaction between CHES1 and SIRT1 not only existed in ERα-positive breast cancer cells but in many ERα-negative cells (Fig. S1B-1D). These results indicated that the association between CHES1 and SIRT1 may exhibit other biological function not completely dependent on ERα. What’s more, previous studies demonstrated that CHES1 may regulated many genes (such as *KLF4* and *c-MYC*) and signaling pathways (such as Wnt/β-catenin) aberrantly active in ERα-negative breast cancer^20,27^. Therefore, the function of CHES1 involved in ERα-negative breast cancer needs further investigation. In order to explore the role of CHES1 in clinical research, we treated MCF7 and T47D cells with three widely used chemotherapy drugs— Cisplatin, Paclitaxel, and Topotecan HCl—and then tested the association between CHES1 and chemotherapeutic sensitivity. The results showed that knockdown of CHES1 had little effect on drug sensitivity (Figure S4A and S4B) but indeed enhanced the proliferation ability of ERα-positive breast cancer cells (Figure S4C). Furthermore, we also found that the overall survival of breast cancer patients who only received chemotherapy treatments have little correlation with the CHES1 expression (Figure S4G). As these chemotherapeutic drugs exhibit their function via inducing apoptosis, an alternative explanation is that CHES1 may not enhance the apoptosis process induced by these drugs. As our study focused on ERα, we also tested the role of CHES1 in endocrine therapy and treated MCF7 cells with Tamoxifen. However, the results indicated that the Tamoxifen sensitivity of MCF7 cells were not affected by shCHES1 (Figure S4D). A possibility is that acetylation of ERα regulated by CHES1 may not affect the hormones sensitivity. Nonetheless, whether CHES1 affects the drug sensitivity of breast cancer cells or not, it needs further extensive drug screening and certification.

## Materials and methods

### Plasmids and reagents

Flag-tagged CHES1 and its control vector plpc-3 × Flag were gifts kindly provided by Dr Gerardo Ferbeyre (Université de Montréal, Canada).The CDS of full-length CHES1, ΔC, and ΔFN mutants were constructed by standard PCR using specific primers and Flag-CHES1 as template. Green fluorescent protein (GFP)-tagged full-length protein and deletion mutants were obtained by PCR and then cloned into GFP-C1 vector at the XhoΙ and EcoRΙ sites. GST-tagged full-length and truncated CHES1 were constructed using similar methods by cloning PCR fragments into pGEX-4T-3 (Amersham Pharmacia, UK). Myc-CHES1 was obtained by using pcDNA3.1-Myc expression vector. Flag-tagged full-length and truncated ERα have been previously described^39^. Flag-SIRT1, Flag-SIRT6, Flag-SIRT7, and GFP-SIRT1 were kindly gifted by Dr Jeong Hoon Kim (Sungkyunkwan University, Korea). shERα has been used in our previous study^40^. CHES1 shRNA expression vectors were constructed by DNA vector-based shRNA synthesis by using the vector pRNATU6.1. The shRNA effects were verified by results of western blotting.

Rabbit polyclonal antibody against ERα (HC-20), mouse monoclonal β-actin (C-2), Fibrillarin (F-6), and SIRT1 (B-10) were obtained from Santa Cruz Biotechnology (Santa Cruz). Rabbit polyclonal antibodies against CHES1 was purchased from Abcam (ab129453) and Abgent (AP19255B). Mouse monoclonal anti-GST antibody (ABN116) was obtained from Millipore. Rabbit polyclonal anti-GFP (GTX113617) antibody was purchased from GeneTex. Rabbit polyclonal anti-c-MYC (SAB4501941), mouse monoclonal anti-Flag (M2) antibodies, and 4-Hydroxytamoxifen (H6278) were purchased from Sigma. Rabbit polyclonal anti-CCND1 (D160236) were obtained from BBI Life Science. Rabbit polyclonal anti-Acetylated lysine (9441) antibody was purchased from CST. ICI 182780 was used as previously described^40^. Rabbit polyclonal anti-Ki67 (27309-1-AP) antibody was purchased from Proteintech. Sirtinol (HY-13515), the specific inhibitor of SIRT1, was obtained from MCE. Cisplatin, Paclitaxel,and Topotecan HCl were purchased from Selleck.

### Cell culture and transfection

HEK293T, COS-7, HeLa, and MDA-MB-231 cells were used in our previous studies^37,55,56^. ZR-75-1, MCF-7, T47D, and SK-BR-3 cells were kindly provided by Dr Wei Cheng of Dalian Medical University. HEK293T, COS-7, HeLa, and MDA-MB-231 cells were cultured in Dulbecco’s modified Eagle’s medium (DMEM, Invitrogen) containing 10% fetal bovine serum (Hyclone) and penicillin–streptomycin (100 U/ml penicillin and 0.1 mg/ml streptomycin). ZR-75-1 cells were cultured in RPMI-1640 medium supplemented with 10% fetal bovine serum and penicillin–streptomycin. T47D cells were cultured in similar complete RPMI-1640 medium in addition with 2 Units/ml bovine insulin. MCF7 cells were cultured in Eagle’s minimum essential medium containing 10% fetal bovine serum, penicillin–streptomycin, and 0.01 mg/ml human recombinant insulin. SK-BR-3 was cultured in McCoy’s 5a medium modified base medium supplemented with 10% fetal bovine serum and penicillin–streptomycin. All cell cultures were incubated at 37 °C in the presence of 5% CO_2_. Transfection assays were carried out using Lipofectamine 2000 (Invitrogen) according to the manufacturer’s instruction.

### Luciferase reporter assay

HEK293T and MCF7 cells transfected with the appropriate plasmids were starved for 24 h with phenol red-free medium containing 2% charcoal-stripped fetal bovine serum (Gibico). Then the medium were replaced by treatment with or without 100 nM E_2_ for 16 h. The cells were then subjected to luciferase reporter assay. Relative luciferase activity was normalized to β-galactosidase and shown as fold changes.

### Immunoprecipitation and western blotting

Cells were collected with the lysis buffer (50 mM Tris-HCl pH 7.4, 150 mM NaCl, 0.1% SDS, 1% NP-40, 0.5% sodium deoxycholate, and protease inhibitor cocktail (Sigma)). After centrifugation at 12,000 × *g* for 15 min at 4 °C, the supernatant was incubated with the desired antibody or with control IgG and protein A-sepharose (GE) at 4 °C for 4 h. After centrifugation at 3000 × *g* for 10 min at 4 °C, the supernatant was abandoned and the precipitate was subjected to wash three time with lysis buffer. Then the pellets was suspended with SDS-polyacrylamide gel electrophoresis (PAGE) 2 × loading buffer, boiling at 100 °C for 5 min and subjected to SDS-PAGE. After electrophoresis, proteins were separated and blotted onto a polvinylidene difluoride membrane (Millipore). Membranes were probed with the specific primary antibody and then peroxidase-conjugated secondary antibodies. The bands were visualized by chemiluminescence.

### Immunofluorescence assay

Immunofluorescence assay was conducted as described previously^57^.

### In vivo tumorigenesis study

All animal experiments and immunohistochemical analysis were approved by the Ethics Committee for Biology and Medical Science of Dalian University of Technology. Human breast cancer xenograft model was constructed as preciously described^57^. Five- to 6-week-old female athymic nude mice (BALB/c mice) were obtained from Animal Experiment Center of Dalian Medical University. Animals were randomly assigned to different groups and each group contained seven animals. Stably transfected tumor cells (1 × 10^6^) in 100 μl of growth medium (mixed with Matrigel (Corning) at 1:1 ratio) were injected subcutaneously. Tumor growth was facilitated by feeding the animals with water containing E_2_ at a concentration of 1 mg/l. Tumor size were measured once five days using a caliper two weeks post injection. Tumor volume were calculated using the standard formula 0.5 × *L* × *W*^2^, where *L* is the longest diameter and *W* is the shortest diameter. Mice were killed after 45 days and the tumor were removed, photographed, and weighed.

### GST-pulldown assay

The GST pulldown assay was carried out as previously described^58^. The GST alone and GST-fusion proteins were expressed in *Escherichia coli* BL21 (Takara) and purified using Pierce GST Spin Purification Kit (Thermo scientific) as per the manufacturer’s instruction. The purified proteins were subjected to immobilized on the Pierce Spin Column and then preyed ERα from MCF-7 cells lysate.

### Cell proliferation assay

Cells were stably transfected with different plasmids and then plated in 96-well plate. Cell proliferation assay were performed using Cell Counting Kit-8 purchased from Solarbio Life Science as manufacture’s instruction. Crystal violet staining assay was carried out by plating 1 × 10^4^ cells in six-well plate. Then cells were grown in with or without E_2_ for a week and subjected to crystal violet staining as previously described.^37^ After staining, wells were washed three times with phosphate-buffered saline and destained with acetic acid, and the absorbance of the crystal violet solution was measured at 590 nm. For cell cycle assay, cells were transfected with the appropriate plasmids and subjected to Flow Cytometry assay with PI staining. Data were collected by FACSCalibur (BD Biosciences). Results were analyzed using ModFit software (BD Biosciences).

### Drug sensitivity assay

Cells were stably transfected with different plasmids and then plated in 96-well plate. Then incubated with different concentration of drugs for indicated time, the cell viability were measured by CCK8 assay.

### Anchorage-independent growth assay

The soft-agar colony culture assay was performed as previously described^57^. Cells (2 × 10^3^) were suspended in 1 ml of 0.3% low-melting-point agarose mixed with 2 × DMEM at 1:1 ratio and plated in triplicate in six-well plate on 1 ml of presolidified 0.7% agarose in the same medium. Then add 1 ml medium to cover the cells and cultured for 3–4 weeks. Cells were stained with MTT (3-(4,5-dimethylthiazol-2-yl)-2,5-diphenyltetrazolium bromide) and the number of colony was counted by using ImageJ program.

### RNA extract and RT-PCR

The cells transfected plasmids indicated were subjected to total RNA extract using RNAiso reagents (Takara). The 2 μg RNA then was reverse transcripted into cDNA using oligo-dT primer. The relative expression of genes was analyzed using standard PCR, the *GAPDH* was set as control. The primers used in RT-PCR were following listed: *GAPDH*, 5′-GGGTTGAACCATGAGAAGT-3′ (forward), 5′-GACTGTGGTCATGAGTCCT-3′ (reverse); *ESR1*, 5′- ACTCGCTACTGTGCAGTGTGCAAT-3′ (forward), 5′- CCTCTTCGGTCTTTTCGTATCCCA-3′ (reverse); *c-MYC*, 5′- AGGGATCGCGCTGAGTATAA-3′ (forward), 5′- TGCCTCTCGCTGGAATTACT-3′ (reverse); *CCND1*, 5′- GCTGCTCCTGGTGAACAAGC-3′ (forward), 5′- AAGTGTTCAATGAAATCGTGCG-3′ (reverse); *pS2*, 5′- ATGGAGAACAAGGTGATCTG-3′ (forward), 5′- CCACAATTCTGTCTTTCACG-3′ (reverse); *CHES1*, 5′- AAATGGAGCGCGGGTCCTGAG-3′ (forward), 5′- GCAGCTGGTGATGCCATTCCT-3′ (reverse).

### Cytoplasmic and nuclear fractionation

The separation of cytoplasmic and nuclear proteins was used Cytoplasmic and Nuclear Fractionation kit obtained from KeyGen BioTECK (Nanjing, China). The process of this assay was conducted as per the manufacturer’s instruction.

### IHC staining assay

IHC staining assay was performed as previously described^37,58^. All the patient species were obtained from Qiqihar Medical University. In addition, 33 slides (17 ERα negative and 16 ERα positive) were incubated with CHES1 and ERα antibodies; the expression levels of CHES1 and ERα were quantified according to their H-scores. The IHC Kit was purchased from ZSGB-BIO (Beijing, China). All individuals who donated the tissues for this study gave their consent in written form. The expression levels of CCND1, c-MYC, and Ki67 in tumors were staining with antibodies as indicated.

### ChIP assay

ChIP assay was carried out as previously described^39^. The lysate of MCF7 cells was immunoprecipitation with antibody against ERα and purified DNA fragment was analyzed by quantitative reverse transcription-PCR. The primers used in the ChIP PCR analysis were 5′-GGCCATCTCACTATGAATCACTTCTGC-3′ (forward) and 5′-GGCAGGCTCTGTTTGCTTAAAGAGCG-3′ (reverse) for *pS2* promoter. The primer for ERE on CHES1 promoter were 5′-AAAGACAAGTGGTGCTATAATTCGT-3′ (forward) and 5′- TTAAGGAGAGAAAACTTCATGAGGC-3′ (reverse).

### Statistical analysis

All the expriments were repeated at least three times. Data were presented as mean ± SDs and Student’s *t*-test (unpaired, two-tailed) was used to compare two groups of independent samples. *P* < 0.05 was considered statistically significant.

## Electronic supplementary material

Supplementary Figure 1

Supplementary Figure 2

Supplementary Figure 3

Supplementary Figure 4

Supplementary Figure 4 continued

Supplementary figure legends

## References

[1] Shang Y, Brown M (2002). Molecular determinants for the tissue specificity of SERMs. Science.

[2] Zhou W, Slingerland JM (2014). Links between oestrogen receptor activation and proteolysis: relevance to hormone-regulated cancer therapy. Nat. Rev. Cancer.

[3] Liang J, Shang Y (2013). Estrogen and cancer. Annu. Rev. Physiol..

[4] Laganière J (2005). Location analysis of estrogen receptor α target promoters reveals that FOXA1 defines a domain of the estrogen response. Proc. Natl Acad. Sci. USA.

[5] Fullwood MJ (2009). An oestrogen receptor α-bound human chromatin interactome. Nature.

[6] Zhang H (2006). The catalytic subunit of the proteasome is engaged in the entire process of estrogen receptor‐regulated transcription. EMBO J..

[7] Métivier R (2003). Estrogen receptor-α directs ordered, cyclical, and combinatorial recruitment of cofactors on a natural target promoter. Cell.

[8] Murakami S, Nagari A, Kraus WL (2017). Dynamic assembly and activation of estrogen receptor alpha enhancers through coregulator switching. Genes Dev..

[9] Lonard DM, Nawaz Z, Smith CL, O’Malley BW (2000). The 26S proteasome is required for estrogen receptor-α and coactivator turnover and for efficient estrogen receptor-α transactivation. Mol. Cell..

[10] Champagne FA (2006). Maternal care associated with methylation of the estrogen receptor-alpha1b promoter and estrogen receptor-alpha expression in the medial preoptic area of female offspring. Endocrinology.

[11] Chen D, Pace PE, Coombes RC, Ali S (1999). Phosphorylation of human estrogen receptor α by protein kinase A regulates dimerization. Mol. Cell. Biol..

[12] Wang C (2001). Direct acetylation of the estrogen receptor alpha hinge region by p300 regulates transactivation and hormone sensitivity. J. Biol. Chem..

[13] Mi YK, Woo EM, Chong YTE, Homenko DR, Kraus WL (2006). Acetylation of estrogen receptor alpha by p300 at lysines 266 and 268 enhances the DNA binding and transactivation activities of the receptor. Mol. Endocrinol..

[14] Sentis S, Le RM, Bianchin C, Rostan MC, Corbo L (2005). Sumoylation of the estrogen receptor alpha hinge region regulates its transcriptional activity. Mol. Endocrinol..

[15] Tremblay AM, Wilson BJ, Yang XJ, Giguère V (2008). Phosphorylation-dependent sumoylation regulates estrogen-related receptor-α and -γ transcriptional activity through a synergy control motif. Mol. Endocrinol..

[16] Moore RL, Faller DV (2013). SIRT1 represses estrogen-signaling, ligand-independent ERα-mediated transcription, and cell proliferation in estrogen-responsive breast cells. J. Endocrinol..

[17] Lam EWF, Brosens JJ, Gomes AR, Koo CY (2013). Forkhead box proteins: tuning forks for transcriptional harmony. Nat. Rev. Cancer.

[18] Myatt SS, Lam EWF (2007). The emerging roles of forkhead box (Fox) proteins in cancer. Nat. Rev. Cancer.

[19] Schuff M (2007). FoxN3 is required for craniofacial and eye development of Xenopus laevis. Dev. Dyn..

[20] Li W (2017). The FOXN3-NEAT1-SIN3A repressor complex promotes progression of hormonally responsive breast cancer. J. Clin. Invest..

[21] Pati D, Keller C, Groudine M, Plon SE (1997). Reconstitution of a MEC1-independent checkpoint in yeast by expression of a novel human fork head cDNA. Mol. Cell. Biol..

[22] Busygina V, Kottemann MC, Scott KL, Plon SE, Bale AE (2006). Multiple endocrine neoplasia type 1 interacts with forkhead transcription factor CHES1 in DNA damage response. Cancer Res..

[23] Whitney EM, Ghaleb AM, Chen X, Yang VW (2006). Transcriptional profiling of the cell cycle checkpoint gene Krüppel-like factor 4 reveals a global inhibitory function in macromolecular biosynthesis. Gene. Expr..

[24] Li Q (2012). MicroRNA-574-5p was pivotal for TLR9 signaling enhanced tumor progression via down-regulating checkpoint suppressor 1 in human lung cancer. PLoS ONE.

[25] Samaan G (2010). Foxn3 is essential for craniofacial development in mice and a putative candidate involved in human congenital craniofacial defects. Biochem. Biophys. Res. Commun..

[26] Scott KL, Plon SE (2005). CHES1/FOXN3 interacts with Ski-interacting protein and acts as a transcriptional repressor. Gene.

[27] Dai Y (2017). Loss of FOXN3 in colon cancer activates beta-catenin/TCF signaling and promotes the growth and migration of cancer cells. Oncotarget.

[28] Chang JT (2005). Identification of differentially expressed genes in oral squamous cell carcinoma (OSCC): overexpression of NPM, CDK1 and NDRG1 and underexpression of CHES1. Int. J. Cancer.

[29] Ying H (2014). Retracted article: Risk miRNA screening of ovarian cancer based on miRNA functional synergistic network. J. Ovarian Res..

[30] Mudduluru G (2015). A systematic approach to defining the microRNA landscape in metastasis. Cancer Res..

[31] Robertson E, Perry C, Doherty R, Madhusudan S (2015). Transcriptomic profiling of Forkhead box transcription factors in adult glioblastoma multiforme. Cancer Genomics Proteomics.

[32] Sun J (2016). The transcription factor FOXN3 inhibits cell proliferation by downregulating E2F5 expression in hepatocellular carcinoma cells. Oncotarget.

[33] Huot G (2014). CHES1/FOXN3 regulates cell proliferation by repressing PIM2 and protein biosynthesis. Mol. Biol. Cell..

[34] Karanth S, Zinkhan EK, Hill JT, Yost HJ, Schlegel A (2016). FOXN3 regulates hepatic glucose utilization. Cell Rep..

[35] Carroll JS (2005). Chromosome-wide mapping of estrogen receptor binding reveals long-range regulation requiring the forkhead protein FoxA1. Cell.

[36] Zou Y (2008). Forkhead box transcription factor FOXO3a suppresses estrogen-dependent breast cancer cell proliferation and tumorigenesis. Breast Cancer Res..

[37] Liu Y (2015). FOXK2 transcription factor suppresses ERα-positive breast cancer cell growth through down-regulating the stability of ERα via mechanism involving BRCA1/BARD1. Sci. Rep..

[38] Shan L (2016). FOXK2 elicits massive transcription repression and suppresses the hypoxic response and breast cancer carcinogenesis. Cancer Cell..

[39] Ao X (2016). Sumoylation of TCF21 downregulates the transcriptional activity of estrogen receptor-alpha. Oncotarget.

[40] Xiao L (2014). Induction of the CLOCK gene by E2-ERα signaling promotes the proliferation of breast cancer cells. PLoS ONE.

[41] Glozak MA, Sengupta N, Zhang X, Seto E (2005). Acetylation and deacetylation of non-histone proteins. Gene.

[42] Spange S, Wagner T, Heinzel T, Krämer OH (2009). Acetylation of non-histone proteins modulates cellular signalling at multiple levels. Int. J. Biochem. Cell. Biol..

[43] Rieber M, Strasberg-Rieber M (2014). p53 inactivation decreases dependence on estrogen/ERK signalling for proliferation but promotes EMT and susceptility to 3-bromopyruvate in ERα+ breast cancer MCF-7 cells. Biochem. Pharmacol..

[44] Asem MS, Steven B, Burkhalter WR, Miller DL, Sharon SM (2016). Wnt5a signaling in cancer. Cancers.

[45] Scott KL, Plon SE (2003). Loss of Sin3/Rpd3 histone deacetylase restores the DNA damage response in checkpoint-deficient strains of Saccharomyces cerevisiae. Mol. Cell. Biol..

[46] Ota H (2006). Sirt1 inhibitor, Sirtinol, induces senescence-like growth arrest with attenuated Ras–MAPK signaling in human cancer cells. Oncogene.

[47] Stender JD (2007). Estrogen-regulated gene networks in human breast cancer cells: involvement of E2F1 in the regulation of cell proliferation. Mol. Endocrinol..

[48] Tang Z (2017). GEPIA: a web server for cancer and normal gene expression profiling and interactive analyses. Nucleic Acids Res..

[49] Györffy B (2010). An online survival analysis tool to rapidly assess the effect of 22,277 genes on breast cancer prognosis using microarray data of 1,809 patients. Breast Cancer Res. Treat..

[50] Shuaiying Cui KEK (2011). . Nuclear receptors TR2 and TR4 recruit multiple epigenetic transcriptional corepressors that associate specifically with the embryonic β-type globin promoters in differentiated adult erythroid cells. Mol. Cell. Biol..

[51] Robinson DR (2017). Integrative clinical genomics of metastatic cancer. Nature.

[52] Kasai H, Allen JT, Mason RM, Kamimura T, Zhang Z (2005). TGF-β1 induces human alveolar epithelial to mesenchymal cell transition (EMT). Respir. Res..

[53] Moses HL, Yang EY, Pietenpol JA (1990). TGF-β stimulation and inhibition of cell proliferation: New mechanistic insights. Cell.

[54] Xu J (2015). 14-3-3ζ turns TGF-β’s function from tumor suppressor to metastasis promoter in breast cancer by contextual changes of Smad partners from p53 to Gli2. Cancer Cell..

[55] Li S (2013). CLOCK is a substrate of SUMO and sumoylation of CLOCK upregulates the transcriptional activity of estrogen receptor-α. Oncogene.

[56] Li S (2016). SUMOylation of PES1 upregulates its stability and function via inhibiting its ubiquitination. Oncotarget.

[57] Bi H (2015). DEC1 regulates breast cancer cell proliferation by stabilizing cyclin E protein and delays the progression of cell cycle S phase. Cell Death Dis..

[58] Zhao F (2015). DACH1 inhibits SNAI1-mediated epithelial–mesenchymal transition and represses breast carcinoma metastasis. Oncogenesis.

