# Supplementary figures and images for: Checkpoint suppressor 1 suppresses transcriptional activity of ERα and breast cancer cell proliferation via deacetylase SIRT1
Source: Cell Death Dis. 2018 May 11;9(5):559. doi: 10.1038/s41419-018-0629-3 (PMC5948204; doi:10.1038/s41419-018-0629-3)

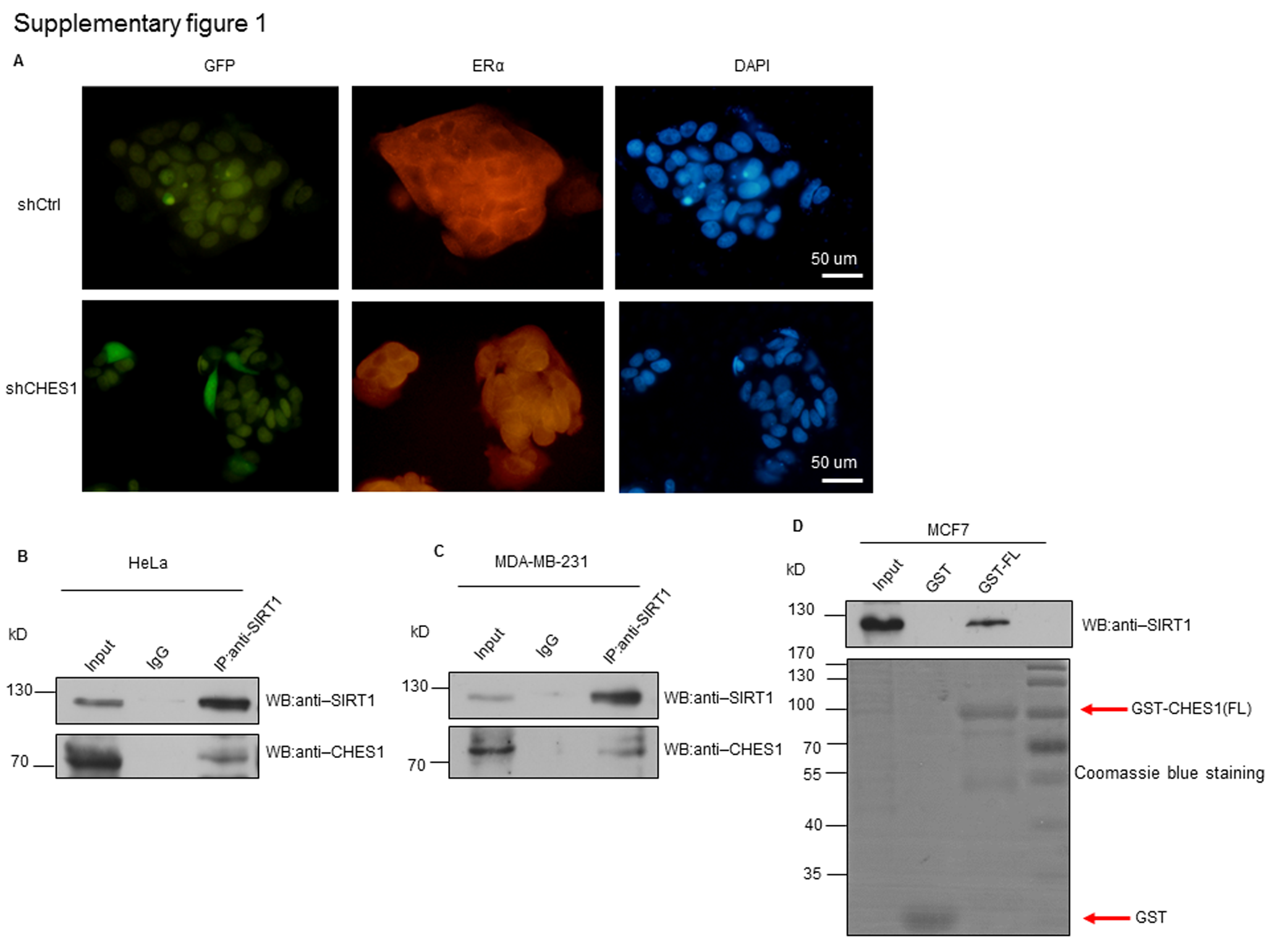

Supplement: Supplementary file 1 — Supplementary Figure 1 [file 41419_2018_629_MOESM1_ESM.tif]

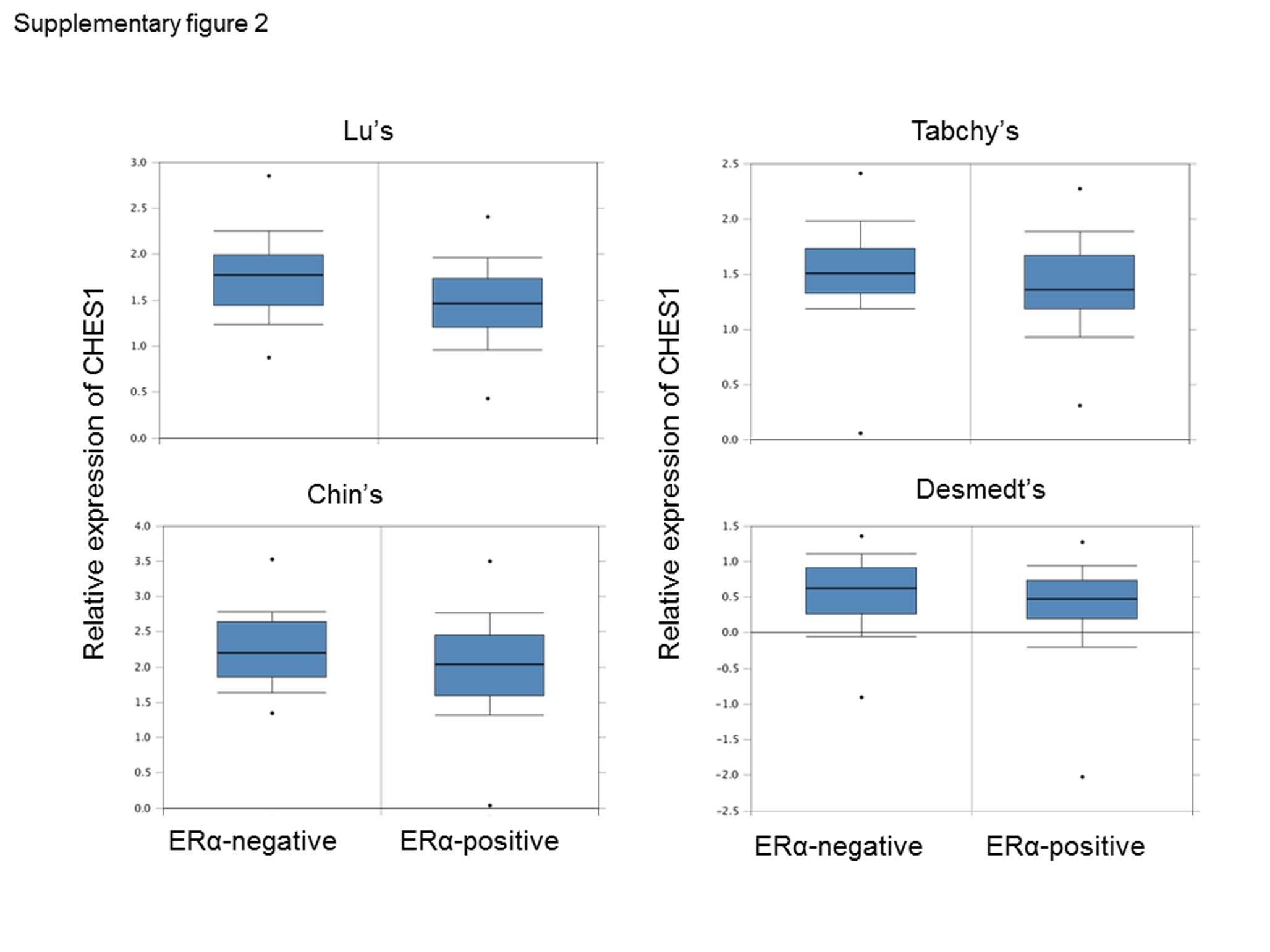

Supplement: Supplementary file 2 — Supplementary Figure 2 [file 41419_2018_629_MOESM2_ESM.tif]

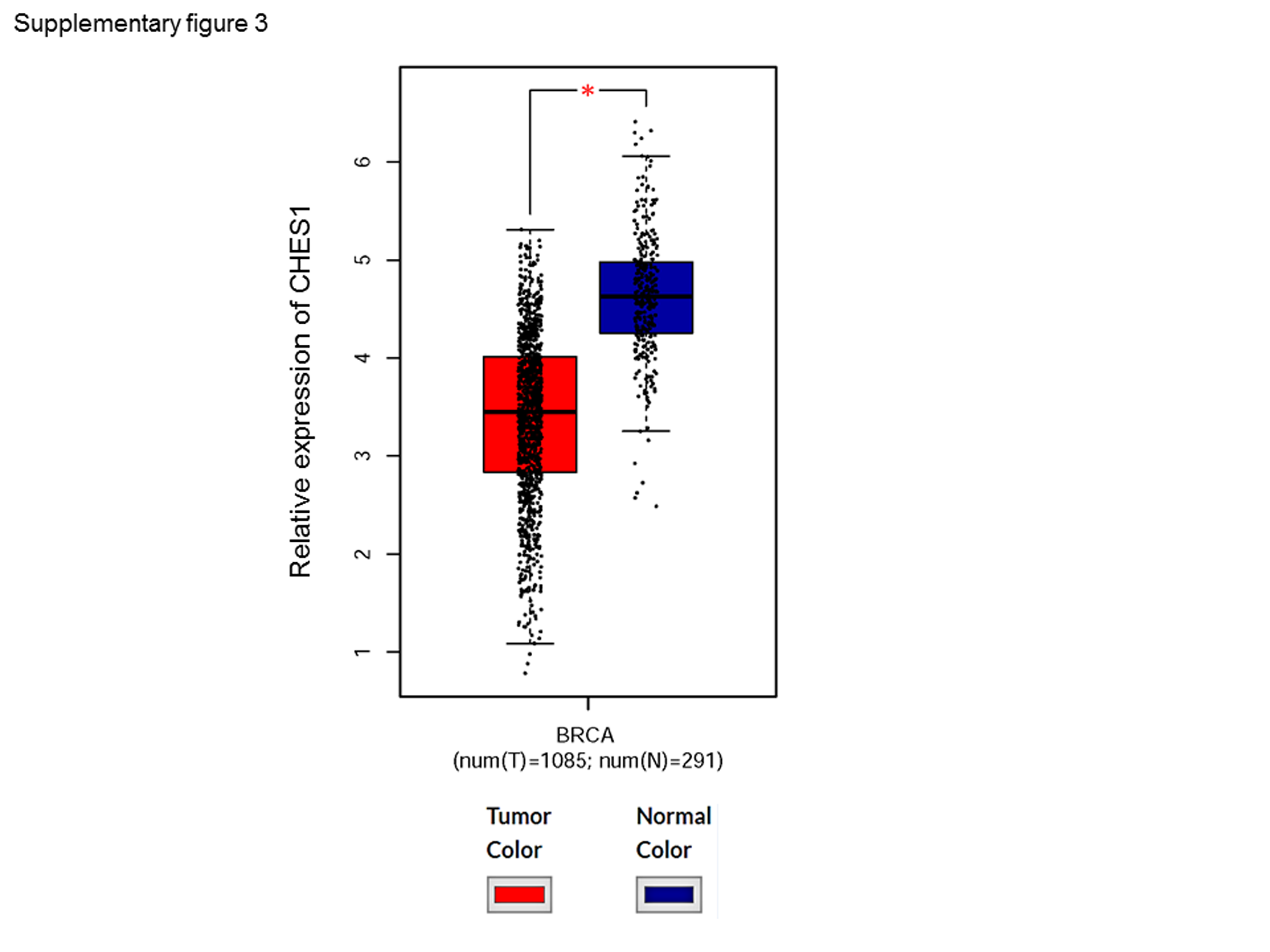

Supplement: Supplementary file 3 — Supplementary Figure 3 [file 41419_2018_629_MOESM3_ESM.tif]

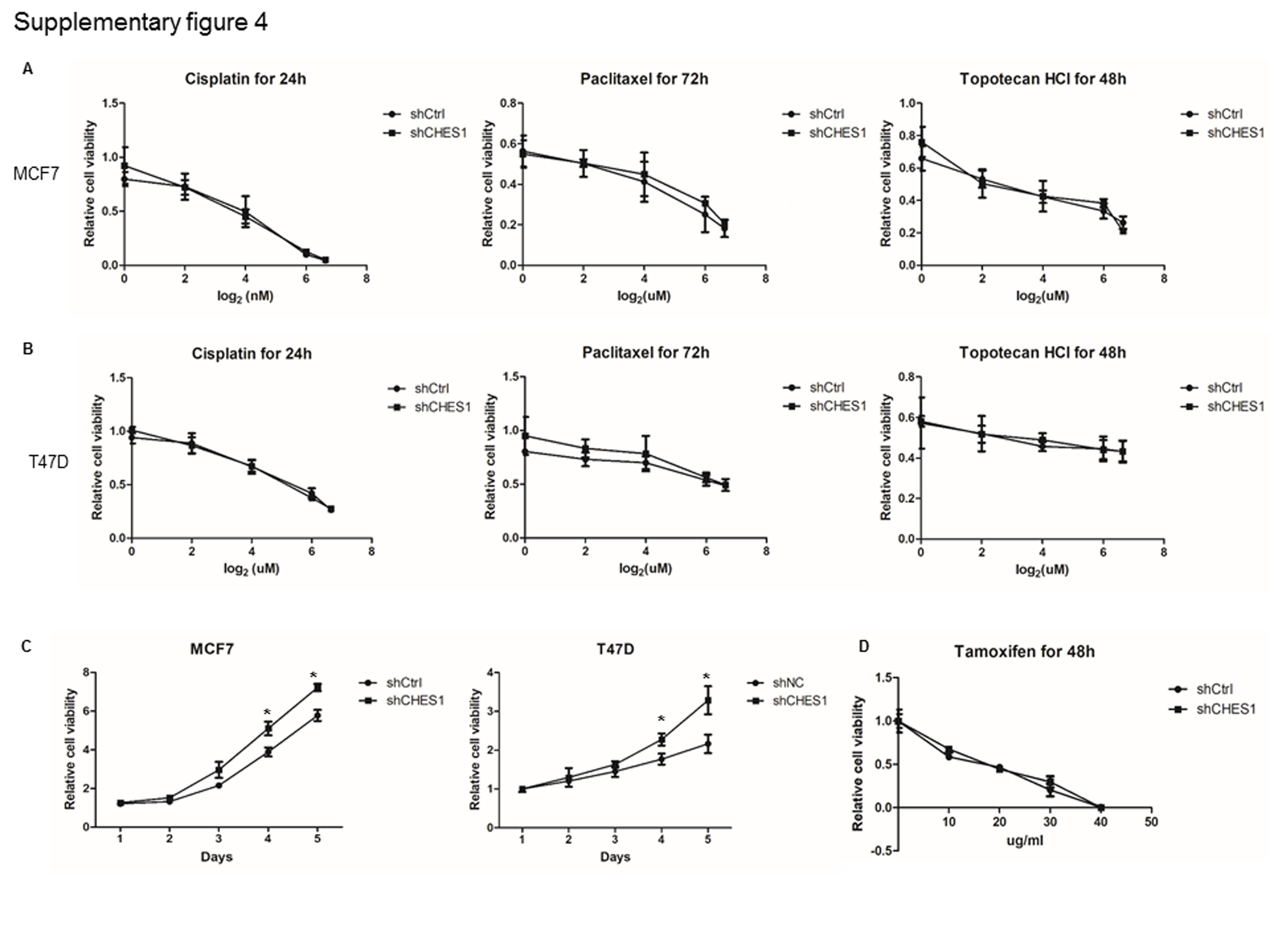

Supplement: Supplementary file 4 — Supplementary Figure 4 [file 41419_2018_629_MOESM4_ESM.tif]

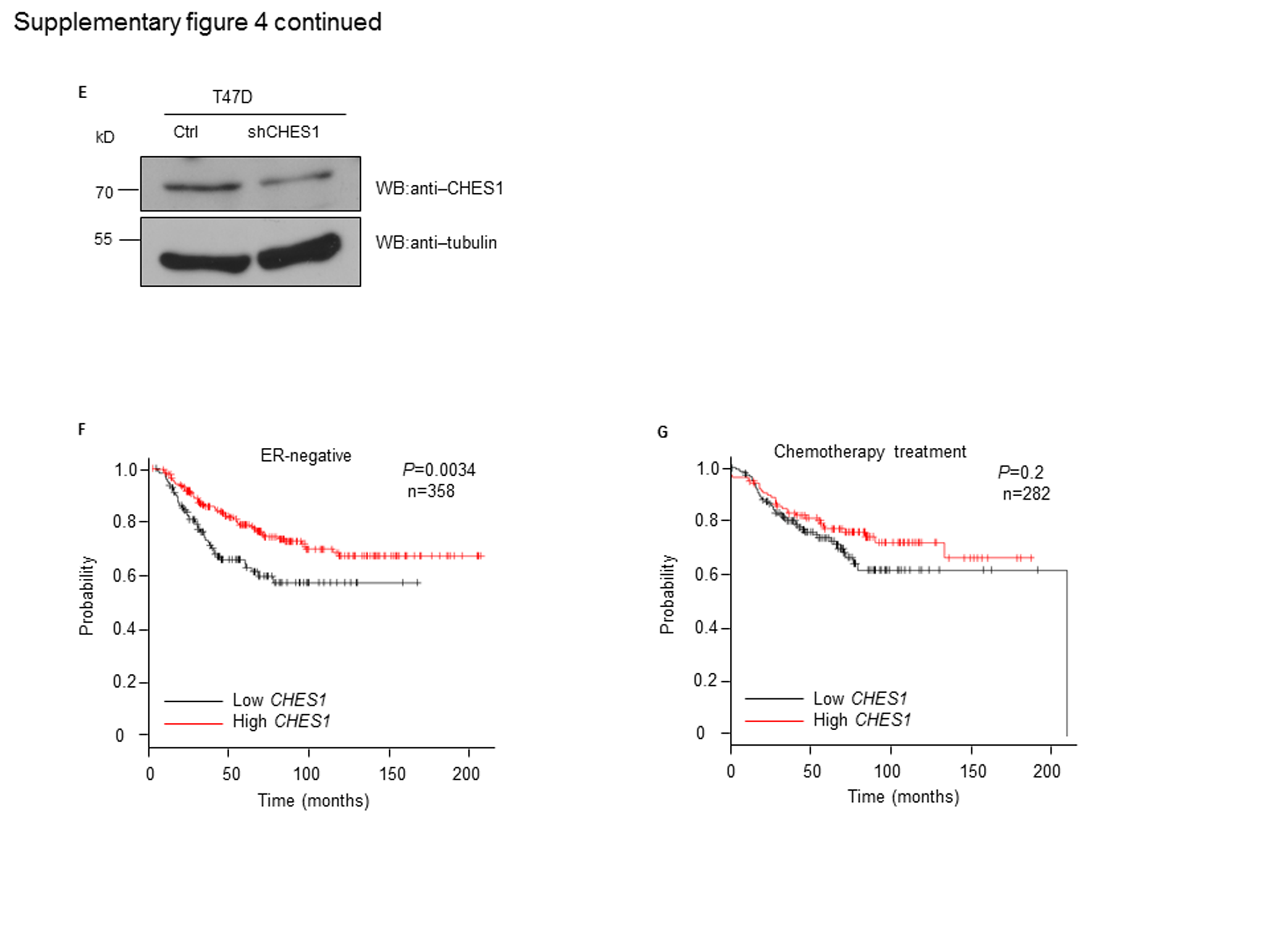

Supplement: Supplementary file 5 — Supplementary Figure 4 continued [file 41419_2018_629_MOESM5_ESM.tif]
