# Supplementary material for: Checkpoint suppressor 1 suppresses transcriptional activity of ERα and breast cancer cell proliferation via deacetylase SIRT1
Source: Cell Death Dis. 2018 May 11;9(5):559. doi: 10.1038/s41419-018-0629-3 (PMC5948204; doi:10.1038/s41419-018-0629-3)
Supplement: Supplementary file 6 — Supplementary figure legends [file 41419_2018_629_MOESM6_ESM.docx]

Figure S1. A. Immunofluorescence assay showed the cellular distribution of endogenous ERα in MCF7 with or without knockdown of CHES1 using shCHES1 (GFP-labeled). B and C. CoIP assay detected the interaction between endogenous CHES1 and SIRT1 in HeLa and MDA-MB-231 cells. D. GST-pulldown assay showed the interaction between purified GST-CHES1 and endogenous SIRT1 in MCF7 cells.

Figure S2. Box plots compared the relative expression level of CHES1 in ERα-positive and ERα-negative breast tumors. The results were analyzed using Microarray data from public datasets Oncomine (<https://www.oncomine.org/resource/login.html>). The differential analysis here is based on the selected datasets.

Figure S3. Box plots compared the relative expression level of CHES1 in breast cancer tumors and normal breast tissues. The results were analyzed using Microarray data from public datasets GEPIA (<http://gepia.cancer-pku.cn/>). The differential analysis here is based on the selected datasets (“TCGA tumors vs TCGA normal + GTEx normal”). The method for differential analysis is one-way ANOVA, using disease state (Tumor or Normal) as variable for calculating differential expression. ‘*’, *P*< 0.05.

Figure S4. A and B. The effect of shCHES1 on chemotherapeutic drug sensibility of MCF7 and T47D cells. C. CCK8 assay tested the effect of shCHES1 on the proliferation ability of MCF7 and T47D cells. D. CCK8 assay examined the Tamoxifen sensitivity of MCF7 cells with or without shCHES1. E. Western blot detected the CHES1 protein in T47D cells with or without knockdown of CHES1 using shRNA. F. The association between CHES1 expression and overall survival of patients with ERα-negative breast cancer. G. The association between CHES1 expression and relapse-free survival of patients only received chemotherapy.
